# Supplementary material for: The Impact of a Family-Based Assessment and Intervention Healthy Lifestyle Programme on Health Knowledge and Beliefs of Children with Obesity and Their Families
Source: Nutrients. 2022 Oct 18;14(20):4363. doi: 10.3390/nu14204363 (PMC9607640; doi:10.3390/nu14204363)
Supplement: Supplementary file 1 [file nutrients-14-04363-s001.zip › nutrients-1877278-supplementary.pdf]

Supplementary File S1. Questions from Whānau Pakari RCT assessment

Knowledge and beliefs about nutrition[a]

	Healthy	Unhealthy	Neither	Don't know
Plain water is:				
All fruit/veges frozen/fresh and canned are:				
Fizzy drinks are:				
Plain milk is:				
Flavoured milk is:				
Takeaways/fast foods are:				
Crisps/chips are:				
Sweets/lollies are:				
Snack bars are:				

What are the benefits of healthy eating?[a]

Knowledge about healthy lifestyles questionnaire[a]

1) What is the amount of physical activity you need to do to stay healthy (minutes per day)?

____

____

2) What are the benefits of physical activity?

____

____

a. Questions are modified from: Clinical Trials Research Unit; Synovate. A National Survey of Children and Young People's Physical Activity and Dietary Behaviours in New Zealand: 2008/09 - Key Findings. 2010.

Supplementary Table S1. Demographic characteristics of Whānau Pakari participants included in analyses of health knowledge over time (food knowledge items) by intervention group.

		BASELINE		12 MONTHS		24 MONTHS		60 MONTHS	
		High-intensity	Low-intensity	High-intensity	Low-intensity	High-intensity	Low-intensity	High-intensity	Low-intensity
**n (%)** [a]		81 (81%)	78	67 (67%)	67	68	53	47	41
**Age (years) M(SD)**		10.7 (3.1)	10.3 (3.2)	11.8 (3.0)	11.3 (3.1)	12.7 (2.9)	12.0 (3.2)	15.4 (3.1)	
**Sex**	**Female**	39 (48.1%)	47 (60.3%)	33 (49.3%)	39 (58.2%)	34 (50.0%)	26 (49.1%)	21 (44.7%)	27 (65.9%)
**Ethnicity** [b]	**New Zealand European**	34 (42.0%)	36 (46.2%)	27 (40.3%)	32 (47.8%)	27 (39.7%)	26 (49.1%)	20 (42.6%)	20 (48.8%)
	**Māori**	38 (46.9%)	35 (44.9%)	31 (46.3%)	28 (41.8%)	33 (48.5%)	22 (41.5%)	23 (48.9%	18 (43.9%)
	**Asian**	5 (6.2%)	1 (1.3%)	5 (7.5%)	1 (1.5%)	5 (7.4%)	1 (1.9%)	2 (4.3%)	-
	**European**	2 (2.5%)	2 (2.6%)	2 (3.0%)	2 (3.0%)	2 (2.9%)	2 (3.8%)	2 (4.3%)	2 (4.9%)
	**Pacific Islander**	2 (2.5%)	2 (2.6%)	2 (3%)	2 (3.0%)	1 (1.5%)	-	-	-
	**Other**	-	2 (2.6%)	-	2 (3.0%)	-	2 (3.8%)	-	1 (2.4%)
**Deprivation quintile** [c]	**1 (least deprived)**	13 (16.0%)	5 (6.4%)	12 (17.9%)	5 (7.5%)	12 (17.6%)	1 (1.9%)	7 (14.9%)	1 (2.4%)
	**2**	15 (18.5%)	9 (11.5%)	12 (17.9%)	9 (13.4%)	12 (17.6%)	9 (17.0%)	8 (17.0%)	6 (14.6%)
	**3**	15 (18.5%)	18 (23.1%)	14 (20.9%)	14 (20.9%)	13 (19.1%)	15 28.3%)	7 (14.9%)	9 (22.0%)
	**4**	15 (18.5%)	24 (30.8%)	12 (17.9%)	23 (34.3%)	13 (19.1%)	16 (30.2%)	13 (27.7%)	14 (34.1%)
	**5 (most deprived)**	23 (28.4%)	22 (28.2)	17 (25.4%)	16 (23.9%)	18 (26.5%)	12 (22.6%)	12 (25.5%)	11 (26.8%)

[a] Percentages represent the proportions of participants assessed at baseline who were re-assessed at a given follow-up.
[b] Prioritised ethnicity.
[c] Based on New Zealand Deprivation Index 2006.

Supplementary Table S2. Demographic characteristics of Whānau Pakari participants included in analyses of physical activity knowledge over time.

		Baseline (All)	12 months	24 months	60 months
**n (%)** [a]		157	144 (60%)	118 (49%)	72 (30%)
**Age (years)**		10.5 [8.1, 13.0]	11.5 [9.3, 13.7]	12.5 [9.9, 14.8]	15.4 [12.4, 18.3]
**Sex**	**Female**	83 (53%)	74 (51%)	63 (53%)	38 (53%)
**Ethnicity** [b]	**New Zealand European**	75 (48%)	70 (49%)	56 (48%)	31 (43%)
	**Māori**	67 (43%)	59 (41%)	49 (42%)	34 (47%)
	**Asian**	6 (4%)	6 (4.3%)	6 (5%)	2 (3%)
	**European**	4 (3%)	4 (3%)	4 (3%)	4 (6%)
	**Pacific Islander**	3 (2%)	3 (2%)	1 (1%)	Nil
	**Other**	3 (1%)	2 (1%)	2 (2%)	1 (1%)
**Deprivation quintile** [c]	**1 (least deprived)**	18 (12%)	18 (13%)	13 (11%)	7 (10%)
	**2**	25 (16%)	24 (17%)	22 (19%)	11 (15%)
	**3**	35 (22%)	33 (23%)	29 (25%)	13 (18%)
	**4**	39 (25%)	35 (24%)	29 (25%)	25 (35%)
	**5 (most deprived)**	40 (26%)	34 (24%)	25 (21%)	16 (22%)

[a] Percentages represent the proportions of participants assessed at baseline who were re-assessed at a given follow-up.
[b] Prioritised ethnicity.
[c] Based on New Zealand Deprivation Index 2006.

Supplementary Table S3. Demographic characteristics of Whānau Pakari participants who were compared to national survey data.

		ALL (BASELINE)	12 MONTHS [a]	24 MONTHS [a]
**5-9 years (food)**				
**n (%)**		98	41 (%)	29 (%)
**Age (years)**		7.6 [5.8, 9.1]	6.8 [5.1, 7.6]	6.3 [5.1, 7.1]
**Sex**	Female	46 (47%)	19 (46%)	16 (55%)
**Ethnicity [a]**	New Zealand European	44 (45%)	21 (51%)	15 (52%)
	Māori	49 (50%)	19 (46%)	14 (48%)
	Asian	1 (1%)	nil	nil
	European	2 (2%)	1 (2%)	nil
	Pacific Islander	2 (2%)	nil	nil
	Other	nil	nil	nil
**Deprivation quintile [b]**	1 (least deprived)	5 (5%)	2 (5%)	2 (7%)
	2	15 (15.3%)	8 (20%)	6 (21%)
	3	25 (26%)	14 (34%)	11 (38)
	4	22 (22%)	6 (15%)	2 (7%)
	5 (most deprived)	31 (32%)	1 (27%)	8 (28%)
**10+ years (physical activity)**				
**n (%)**		134	98 (%)	89 (%)
**Age (years)**		13.0 [11.4, 14.3]	10.4 [10.4, 13.3]	11.2 [9.6, 13.2]
**Female**		76 (57%)	52 (53%)	47 (53%)
**Ethnicity [b]**	New Zealand European	62 (46%)	45 (46%)	41 (46%)
	Māori	56 (42%)	39 (40%)	35 (39%)
	Asian	5 (4%)	6 (6%)	6 (7%)
	European	4 (3%)	3 (3%)	4 (5%)
	Pacific Islander	4 (3%)	3 (3%)	1 (1%)
	Other	3 (2%)	2 (2%)	2 (2%)
**Deprivation quintile [c]**	1 (least deprived)	17 (13%)	16 (16%)	11 (12%)
	2	22 (16%)	15 (15%)	16 (18%)
	3	23 (17%)	18 (18%)	18 (20%)
	4	38 (28%)	28 (29%)	27 (30%)
	5 (most deprived)	34 (25%)	21 (21%)	17 (19%)

[a] Percentages represent the proportions of participants assessed at baseline who were re-assessed at a given follow-up.
[b] Prioritised ethnicity.
[c] Based on New Zealand Deprivation Index 2006.

Supplementary Table S4. Number and proportion of Whānau Pakari participants who correctly answered items in the health knowledge questionnaire at baseline and follow-up assessments, by trial randomisation group.

	High-intensity intervention		Low-intensity control		High-intensity intervention		Low-intensity control		High-intensity intervention		Low-intensity control	
	**Baseline**	**12 months**	**Baseline**	**12 months**	**Baseline**	**24 months**	**Baseline**	**24 months**	**Baseline**	**60 months**	**Baseline**	**60 months**
**HEALTHY EATING**												
**n**	68	68	61	61	58	58	48	48	43	43	38	38
**Healthy**												
Plain water	65 (96%)	65 (96%)	52 (85%)	59 (97%)	56 (97%)	58 (100%)	40 (85%)	47 (100%)*[a]	40 (95%)	42 (100%)[b]	29 (83%)	35 (100%)*[c]
Plain milk	49 (72%)	62 (91%)**	42 (69%)	49 (80%)	41 (71%)	55 (95%)*	35 (73%)	40 (83%)	32 (74 %)	38 (88%)	27 (73%)	29 (78%)[d]
All fruit and vegetables	58 (85%)	67 (99%)*	52 (85%)	56 (92%)	49 (84%)	56 (97%)*	42 (88%)	45 (94%)	36 (84%)	41 (95%)	32 (86%)	36 (97%)[d]
**Unhealthy**												
Flavoured milk	42 (62%)	45 (66%)	36 (59%)	44 (72%)	35 (60%)	42 (72%)	29 (60%)	45 (94%)***	27 (63%)	38 (88%)*	24 (63%)	33 (87%)*
Fizzy drinks	61 (90%)	67 (99%)	55 (90%)	58 (95%)	52 (90%)	57 (98%)	44 (92%)	48 (100%)	39 (91%)	43 (100%)	35 (92%)	37 (97%)
Takeaways and fast foods	59 (87%)	66 (97%)*	47 (77%)	57 (93%)*	51 (88%)	55 (95%)	38 (79%)	47 (98%)**	38 (88%)	42 (98%)	30 (79%)	34 (89%)
Crisps and chips	60 (88%)	64 (94%)	45 (74%)	56 (92%)**	52 (90%)	53 (91%)	36 (75%)	45 (94%)*	38 (88%)	42 (98%)	29 (76%)	36 (95%)*
Sweets and lollies	65 (96%)	67 (99%)	54 (89%)	59 (97%)	56 (97%)	56 (97%)	43 (90%)	48 (100%)	42 (98%)	43 (100%)	36 (95%)	38 (100%)
Snack bars	23 (34%)	40 (59%)**	27 (44%)	22 (36%)	19 (33%)	34 (59%)**	24 (50%)	31 (65%)	15 (35%)	27 (63%)*	17 (45%)	20 (53%)
**PHYSICAL ACTIVITY**												
**n**	67	67	61	61	57	57	47	47	40	40	32	32
**≥60 min/day**	20 (30%)	56 (84%)***†	24 (39%)	36 (59%)*	18 (32%)	46 (81%)***	17 (36%)	34 (72%)***	10 (25%)	31 (78%)***†	12 (38%)	17 (53%)

Data are n (%), corresponding to the number and proportion of participants with correct responses at a given assessment.
Baseline data for comparison are only provided for those participants with corresponding data at a given follow-up assessment.
*p<0.05, **p<0.01, and ***p<0.001 for statistically significant within-group differences from baseline, derived from McNemar's tests.
†p<0.05 for a statistically significant difference between groups at p<0.05, based on a two-sample McNemar's tests
[a] n=47; [b] n=42; [c] n= 35; and [d] n=37.
